# IntAct App: a Cytoscape application for molecular interaction network visualization and analysis

**DOI:** 10.1093/bioinformatics/btab319

**Published:** 2021-05-07

**Authors:** Eliot Ragueneau, Anjali Shrivastava, John H Morris, Noemi del-Toro, Henning Hermjakob, Pablo Porras

**Affiliations:** European Bioinformatics Institute (EMBL-EBI), European Molecular Biology Laboratory, Wellcome Genome Campus, Hinxton, Cambridgeshire CB10 1SD, UK; European Bioinformatics Institute (EMBL-EBI), European Molecular Biology Laboratory, Wellcome Genome Campus, Hinxton, Cambridgeshire CB10 1SD, UK; Resource for Biocomputing, Visualization, and Informatics, Department of Pharmaceutical Chemistry, University of California, San Francisco, CA 94158 2517, USA; European Bioinformatics Institute (EMBL-EBI), European Molecular Biology Laboratory, Wellcome Genome Campus, Hinxton, Cambridgeshire CB10 1SD, UK; European Bioinformatics Institute (EMBL-EBI), European Molecular Biology Laboratory, Wellcome Genome Campus, Hinxton, Cambridgeshire CB10 1SD, UK; European Bioinformatics Institute (EMBL-EBI), European Molecular Biology Laboratory, Wellcome Genome Campus, Hinxton, Cambridgeshire CB10 1SD, UK

## Abstract

**Summary:**

IntAct App is a Cytoscape 3 application that grants in-depth access to IntAct’s molecular interaction data. It build networks where nodes are interacting molecules (mainly proteins, but also genes, RNA, chemicals…) and edges represent evidence of interaction. Users can query a network by providing its molecules, identified by different fields and optionally include all their interacting partners in the resulting network. The app offers three visualizations: one only displaying interactions, another representing every evidence and the last one emphasizing evidence where mutated versions of proteins were used. Users can also filter networks and click on nodes and edges to access all their related details. Finally, the application supports automation of its main features *via* Cytoscape commands.

**Availability and implementation:**

Implementation available at https://apps.cytoscape.org/apps/intactapp, while the source code is available at https://github.com/EBI-IntAct/IntactApp.

## 1 Introduction

IntAct is an open-source molecular interaction database which captures experimental evidence from the literature in high detail (Orchard *et al.*, 2014), following the deep curation model developed in the IMEx Consortium (Porras *et al.*, 2020). One of the challenges faced by IntAct is to provide efficient ways to access and display the rich detail of its data. Cytoscape is an answer to this issue, as it grants biologists unparalleled flexibility to visualize, manipulate and analyse networks, especially through the many tools available as Cytoscape apps (Shannon *et al.*, 2003).

Different tools, such as PSICQUIC (del-Toro *et al.*, 2013) or the BioGateway App (Holmås *et al.*, 2020) already provide access to

IntAct’s molecular interactions. However, they do not represent the full depth of detail in IntAct data as they are meant to integrate other databases as well, and therefore use a shallow model of the available data.

IntAct App aims to provide full access to the different layers of IntAct, ensuring their readability by offering different predefined styles, nested navigation and filtering capabilities on multiple levels.

## 2 Features

Users can build IntAct networks by querying for a set of molecule names, identifiers or descriptors. These will define the network participants, visualizing interactions between them and, optionally, all interacting partners. Ambiguous symbols or identifiers, matching more than one molecule in IntAct, can be dealt with thanks to a preview panel displaying all matches found per search term. IntAct App provides two query modes that are distinct in the way this ambiguity is dealt with:

- ‘*Exact query*’, to minimize the possibility of ambiguity. It should be used when the user has precise, unambiguous identifiers. It requires complete identifiers or gene names.
- ‘*Fuzzy search*’, a broader search to collect everything associated with the given terms. It also allows partial matches of names and descriptions for the target molecules.

IntAct App provides three styling options, or ‘views’, of its networks. The ‘Evidence’ view represents every evidence (one interaction observed by one technology in one publication) as a distinct edge. The ‘Summary’ view collapses all interaction evidence between each pair of molecules into a single edge. Finally, the ‘Mutation’ view also separates each evidence but highlights edges in which one of the participants is mutated.

To highlight cross-species interactions, molecules are styled according to the species they belong to with a palette based on their taxonomy.

IntAct App allows style customization via the interactive legend, so that users can reassign node color according to their preference.

Selection of IntAct network elements triggers the display of all information relative to them in the application panel. Available data are fully described in the user guide (https://ebi-intact.github.io/IntActApp/).

The drop down information menus on this panel can also be used to filter the data according to different criteria, such as the confidence score associated with the interactions (Villaveces *et al.*, 2015), interaction types, detection methods or molecule species.

IntAct App also supports the creation of sub networks, Cytoscape session saves and its core features are available via command line, thus allowing automation and scripting access (https://ebi-intact.github.io/IntActApp/automation_support).

## 3 Use case

Angiotensin-converting enzyme 2 (ACE2) is a crucial protein in the COVID-19 pandemic as it has been identified as the entrance receptor for the surface spike glycoprotein (S) of SARS-CoV-2 (Hoffmann *et al.*, 2020). In IntAct App, a fuzzy search with the term ‘ACE2’ builds a network with eight orthologs of ACE2, including the human processed form. Two yeast hits, which map to a completely different protein sharing the same symbol, can be easily discarded through the preview panel.

**Fig. 1.**
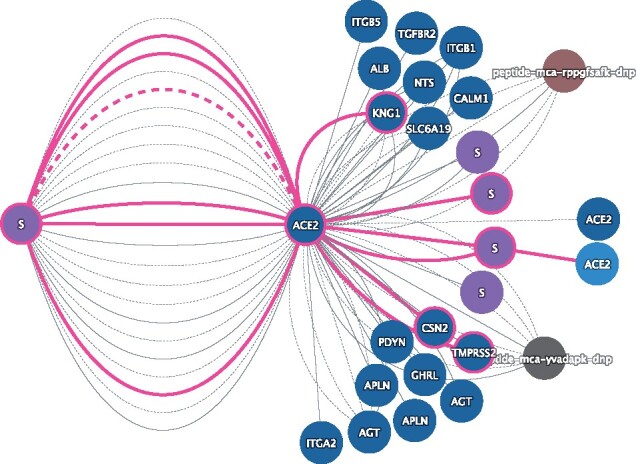
ACE2 fuzzy search filtered result on Mutation view. Each interaction evidence is shown as a separate edge. Molecules and interactions with mutation information are highlighted in pink. SARS-CoV data has been hidden to focus on SARS-CoV-2

The human ACE2 (Q9BYF1) shows evidence of interaction with 7 Spike proteins coming from different viruses, including human SARS-CoV (MI Score: 0.98) and SARS-CoV-2 (MI Score: 0.99). Shifting to ‘Evidence’ view allows to see the full extent of evidence behind these well-characterized interactions. The ‘Mutation’ view (Fig. 1) tells that mutations on both Spike and ACE2 might have an impact on their binding. Several of these mutations were found to increase the interaction strength, among which N501T, a mutation of the Spike protein on the same position as N501Y, which is found on the highly transmissible VOC 202012/01 strain (Leung *et al.*, 2021) and other emerging strains. The information recorded in IntAct highlights the importance of the N501 residue in Spike-ACE2 binding and could help refine current hypothesis about the increased transmissibility of some of these strains.

## 4 Conclusion

IntAct App allows for the representation of molecular interaction networks derived from the IntAct database, providing unprecedented access to the full level of experimental detail featured in IntAct records. For the first time, users can easily navigate and filter details about interaction detection methods, experimental hosts, binding regions or mutations affecting interaction outcome, using customizable and flexible styling options to focus on different aspects of the data. The app is also meant to complement IntAct’s website, providing support for representation of large networks.

Planned development will focus on providing advanced query types, for example allowing the construction of full interactomes, and exploring integration capabilities with other apps and functionalities of Cytoscape.
